# Chandipura virus outbreak: a growing public health crisis that demands immediate action

**DOI:** 10.1097/MS9.0000000000002841

**Published:** 2025-01-09

**Authors:** Huda Adnan, Izere Salomon, Maryam Ahmed

**Affiliations:** a Jinnah Sindh Medical University, Karachi, Pakistan; b University of Rwanda College of medicine and Health sciences, Kigali, Rwanda

Chandipura virus (CHPV) is a member of the *Rhabdoviridae* family and *vesiculovirus* genera[1], is an arthropod-born virus first discovered in 1966 by Bhatt and Rodriguez at the virus research Centre in Pune, the discovery was deemed accidental while investigating patients suffering from fever in Chandipura village, hence the name CHPV. The transmission of the virus usually occurs by sandflies (*Phlebotomus* spp), ticks, and mosquitoes (*Aedes egypti*) with clinical manifestations including an influenza-like illness followed by abdominal pain, vomiting, impaired neurological functioning including encephalitis and altered consciousness (Fig. 1)[2].

**Figure 1.**
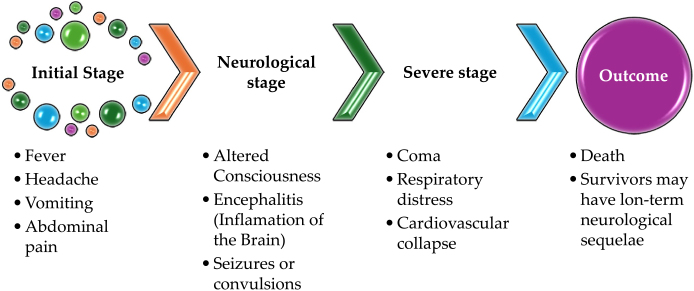
Symptoms and disease progression in Chandipura virus infection.[2]

CHPV causes neuronal death via the Fas-mediated extrinsic apoptotic pathway, which activates caspase 3 and 8. The virus exhibits diversity in its genetics by forming distinct clades in West Africa and India and has the potential for human spillover due to adaptation in codon usage[3]. CHPV infiltrates the central nervous system (CNS) via disruption of the blood–brain barrier (BBB) and utilizing neuronal retrograde transport. It triggers cellular stress and leads to the production of reactive oxygen species. Toll-like receptors 4, which are a part of the innate immune response are an important part of the pathogenesis and result in the production of nitric oxide (NO) and pro-inflammatory cytokines, thereby contributing to the progression of the disease. CHPV enters the CNS via the hematogenous route and infects immune cells including Langerhans, macrophages, monocytes, and dendritic cells. These cells then transfer the virus to endothelial cells and epithelial cells. Neurotropic viruses like CHPV cause increased permeability of the BBB. After breaching the BBB, the infected monocytes differentiate into dendritic cells and macrophages which produce tumor necrosis factor and NO thereby causing neuroinflammation[3].

The CHPV is endemic in India with previous outbreaks occurring during the monsoon season in the western, central, and southern parts of India. The virus mostly affects children under the age of 15 with symptoms including a febrile illness that may progress to coma, convulsions, and even death, it may lead to increased mortality within 42–72 hours after symptom onset typically presenting with acute encephalitis syndrome (AES). According to the Ministry of Health and Family Welfare of the Government of India between early June and 15 August 2024, the number of cases has steadily increased with up to 245 AES cases, 64 of them being confirmed to be from CHPV making it the largest outbreak in the past 20 years (Table 1)[4].

**Table 1:** Epidemiological overview of Chandipura virus outbreak in India (June—August 2024)[4]

Category	Details	Category	Details
Total AES cases	245 cases	Confirmed CHPV Cases	64 cases
CFR for AES	33% (82 deaths)	CFR for CHPV	Historically 56–75%
Geographical distribution	Gujarat: 61,	Predominant age group	Children under 15 years
	Rajasthan: 3		
Primary vector	Phlebotomus papatasi (Sandfly)	Outbreak onset	Early June 2024
Peak outbreak period	July 2024	Decline in new cases	Observed since 19 July 2024
Notable previous outbreaks	Andhra Pradesh, 2003: 329 cases, 183 deaths		
	Maharashtra: 114 deaths		
	Gujarat: 24 Deaths		

The largest outbreak of this virus happened in 2003–2004 in Gujarat, Andhra Pradesh (AP), and Maharashtra with the total death count being reported as 322 which includes 183 in AP, 114 in Maharashtra, and 24 in Gujarat^[5,6]^. The gold standard test for the detection of neutralizing antibodies against CHPV is the plaque reduction neutralizing test; however, it is time intensive and the reading is subjective therefore micro neutralization enzyme-linked immunosorbent assay is used which has a shorter turnaround time[7]. The management of this disease is usually symptomatic. Medications including non-steroidal anti-inflammatory drugs (NSAID) and aspirin should be avoided as they might worsen the disease due to their potential adverse effects. Treatment is usually focused on managing complications such as increased intracranial pressure, seizures, and hyponatremia. Currently, there is no vaccine available and no specific antiviral medications are effective against this infection[2].

Since it is a vector-controlled disease insecticide spraying and fumigation should be done to control the spread of infection especially with the monsoon season approaching. Large areas with water accumulation should be cleared immediately as they serve as breeding hubs for mosquitos. Awareness regarding the smearing of cow dung on the floor and walls of houses in endemic areas should be provided as cow dung serves as feed for the sand fly larvae[8]. Poor housing conditions and improper waste management may cause an increase in the vector population; therefore, the government needs to address the situation within due time. Health awareness campaigns should be started to equip the public with the required knowledge necessary to combat the disease. Surveillance efforts should be started in high-risk populations, i.e. children younger than 15 years presenting with fever and neurological symptoms.

The outburst of CHPV, if not contained will lead to a critical health emergency especially among children, as happened previously in the 2003–2004 outbreak, where case fatalities ranged from 55% to 75%. The severe fatality rate will create a strain on the healthcare system and require more resources for the treatment and management of cases[8]. Families affected by the virus struggle financially to pay hefty medical bills along with medicines. The constant need for healthcare equipment strains the local economy as it diverts funds from other essential services. CHPV affects people from lower socioeconomic backgrounds, as seen in the outbreaks in Bihar, due to poor hygiene strategies. This economic burden of healthcare costs and loss of labor can overly strain the economy of a country with lasting negative impacts[9].

Previously, during the outbreak of CHPV in 2003–2004, high morality and mortality rates were observed in central India due to several mistakes. One of the considerable issues was the rapid progression of the disease, with symptoms of encephalitis within 24 hours. The acute nature of illness often resulted in delays in diagnosis and treatment, which contributed to high mortality rates[10]. Secondly, the rapid mechanism of action of CHPV remained unknown at that time, which complicated the effective treatment strategies. As recent studies have observed, the virus’s rapid destruction of neuronal cells causes severe damage in no time, leaving a brief period for effective medical intervention[11]. Since there are no specific antiviral treatments or vaccines available only supportive treatments can improve the disease outcome therefore basic supportive healthcare facilities should be provided by the government to decrease the progression of the disease[4].

Cases like the rare occurrence of CHPV infection with hemorrhagic complications in Gujarat have highlighted the virus’s severity, drawing significant media attention. This increased public awareness is crucial for minimizing the risks and complications associated with CHPV, emphasizing the importance of early detection and treatment[12].

In conclusion, the CHPV outbreak in India requires immediate action and comprehensive strategies to contain the virus. The outbreak sheds light on the vulnerabilities of the public health system in India and the need for long-term strategies to prevent similar outbreaks from happening in the future.

## Data Availability

No other datasets were generated during and/or analyzed during the current study. All the information is available with the manuscript.
